# From acute to chronic: the PREVENT protocol for understanding pain progression after emergency department visits

**DOI:** 10.1017/S146342362510042X

**Published:** 2025-09-11

**Authors:** Michael Ray, Andrew C. Meltzer, Timothy C. McCall, Keith Meldrum

**Affiliations:** 1 Department of Emergency Medicine, The George Washington University School of Medicine and Health Sciences, Washington, District of Columbia, USA; 2 Department of Clinical Research and Leadership, The George Washington University School of Medicine and Health Sciences, Washington, District of Columbia, USA; 3 Unaffiliated, Kelowna, BC, Canada

**Keywords:** Acute pain, emergency medicine, low back pain, neck pain, prevention

## Abstract

**Aim:**

The Pain Recognition and Evaluation to Validate Effective Neck and back Treatment (PREVENT) study aims to identify cognitive, behavioral, and treatmentrelated predictors of chronic musculoskeletal pain (CMP) development following emergency department (ED) care for acute neck or back pain after trauma.

**Background:**

CMP is a leading cause of global disability, yet early risk factors for its development remain poorly characterized, particularly in ED settings. This prospective observational study will recruit 246 adult patients presenting with acute (≤ 4 weeks) neck or back pain after a recent trauma. Pain beliefs – measured using pain and attitude questionnaires – serve as the primary independent variable. Mediating variables include catastrophic thinking, fear-avoidance behaviors, low physical activity, poor recovery expectations, and low self-efficacy for pain management. Covariates include demographics, social determinants of health, mental health disorders, and high-risk substance use. The primary outcome is the presence of CMP at six months, defined as pain on most or every day for at least three months. Participants will complete follow-ups at 1, 3, and 6 months. Multivariable logistic regression, mediation analyses, and interaction testing will explore effects of pain beliefs on CMP development. As a secondary aim, a subset of participants will complete Think Aloud cognitive interviews to assess response process validity for the Neck Pain Attitudes Questionnaire (Neck-PAQ), a region-specific adaptation of the Back Pain Attitude Questionnaire, analyzed using a deductive content analysis framework.

**Discussion:**

This study is among the first to investigate the cognitive and behavioral predictors of pain chronification in the ED. Ethical approval has been obtained from The George Washington University Institutional Review Board. Findings will inform the design of targeted, ED-based screening and intervention strategies, including adaptation of a pain-specific Screening, Brief Intervention, and Referral to Treatment (SBIRT) model. Results will be disseminated through peer-reviewed publications, conferences, and stakeholder engagement.

## Introduction

Chronic musculoskeletal pain (CMP) is the leading cause of disability worldwide, affecting millions annually and imposing an economic burden of $560–635 billion in the U.S. alone (Vos *et al.*, 2020; Gaskin & Richard, 2012). CMP, characterized by persistent or recurrent pain in the muscles, bones, joints, or tendons, most commonly affects the neck and lower back regions (Treede *et al.*, 2015; World Health Organization, 2022). Emergency departments (EDs) frequently serve as the initial point of care for individuals experiencing either acute or chronic pain, with musculoskeletal pain-related complaints representing the fourth most common reason for ED visits in 2021 (9.5 million cases) (Centers for Disease Control and Prevention, 2023).

Despite the ED’s important role in addressing pain across the acute-chronic continuum, limited research has examined ED-specific risk factors contributing to the transition from acute to chronic pain, and few interventions have been systematically implemented to prevent this trajectory. In the general population, the annual incidence of chronic pain is 6.3% (Nahin *et al.*, 2023), but among ED patients with acute pain, the risk of chronic pain transition is substantially higher, ranging from 12%–27% (Beaudoin *et al.*, 2020; Office of the Assistant Secretary for Planning and Evaluation, 2021; Bourassa *et al.*, 2020; Barnett *et al.*, 2017), underscoring the ED as a critical setting for early identification and prevention. Prior ED-based studies have identified predictors such as pain intensity, body region, and opioid use (Beaudoin *et al.*, 2020; Office of the Assistant Secretary for Planning and Evaluation, 2021; Bourassa *et al.*, 2020; Barnett *et al.*, 2017), but the role of maladaptive pain beliefs, kinesiophobia, pain catastrophizing, and guideline-discordant care remains largely unexplored. Evidence from post-surgical and outpatient populations has established that these psychological factors and guideline-discordant care (e.g., excessive imaging) are associated with an increased risk of developing chronic pain (Theunissen *et al.*, 2012; Stevans et al., 2020), and this is further supported by observational and conceptual studies linking these factors to pain persistence, negative affect, and pain-related disability (Rogers & Farris, 2022; Coninx *et al.*, 2025; Ray *et al.*, 2022; Keller *et al.*, 2023; Brown *et al.*, 2020; Vivekanantha *et al.*, 2023). However, their potential value in ED-based screening and intervention remains untested.

The overarching goal of this study is to determine whether maladaptive pain beliefs, fear-avoidance behaviours, catastrophizing, and guideline-discordant care contribute to the transition from acute to chronic pain among ED patients with acute post-traumatic musculoskeletal pain. We hypothesize that individuals who transition to CMP will exhibit greater psychological distress (e.g., anxiety, depression, pain catastrophizing), misinformed pain beliefs (e.g., kinesiophobia), higher rates of non-prescribed psycho-active substance use (e.g., alcohol, marijuana), and distinct patterns of ED-based pain management (e.g., increased imaging and pharmacologic interventions).

**Figure 1.**
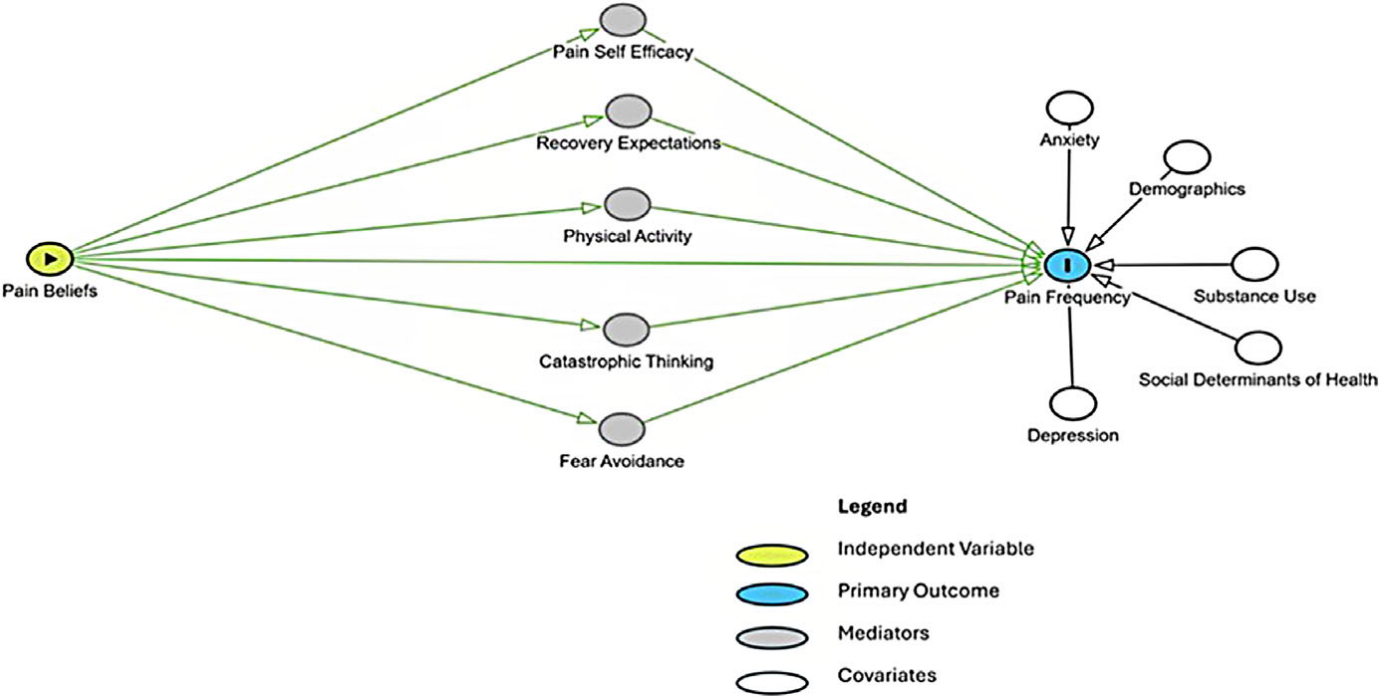
Cognitive Risk Model for Chronic Musculoskeletal Pain. This directed acyclic graph (DAG) illustrates hypothesized pathways through which pain beliefs influence the development of chronic musculoskeletal pain (pain frequency) following an ED visit. Psychological and behavioural mediators (grey), confounding variables (white), and primary outcome (blue) are shown, with arrows representing theorized causal relationships.

To guide our study design and selection of predictors, we developed a conceptual framework titled the Cognitive Risk Model for CMP (Figure 1). Grounded in the fear-avoidance and biopsychosocial models (Vlaeyen *et al.*, 2016; Engel, 1977), this framework posits that maladaptive pain beliefs influence chronic pain development through psychological and behavioural pathways. It is further informed by emerging affordance-based theories of pain, which suggest that persistent pain alters perceived opportunities for action (i.e. affordances), reinforcing avoidance behaviours (Coninx *et al.*, 2025). This theoretical lens highlights the importance of addressing cognitive and behavioural responses to acute pain early in the care trajectory. The study aims to test this framework in an ED setting, with the goal of identifying individuals at heightened risk of chronic pain development and informing future early intervention strategies.

## Methods and analysis

### Conceptual framework & analytic rationale

Building upon the framework described above, the Cognitive Risk Model for CMP conceptualizes pain beliefs and attitudes as the primary independent variable, with the presence of CMP as the primary outcome, and functional status and pain intensity as secondary outcomes. By applying this theoretical lens, our study aims to identify how pain beliefs and behavioural responses constrain meaningful action and contribute to pain persistence. This model informed the selection of variables, timing of follow-up assessments, and the structure of our analytic approach. Specifically, it supports the inclusion of psychosocial and behavioural mediators (e.g., catastrophizing, fear-avoidance, physical activity, pain self-efficacy, recovery expectations), and contextual modifiers (e.g., substance use, social determinants of health, mental health conditions), and pathway modelling to explore both direct and indirect effects.

The study design was further refined and strengthened through consultation with ED clinicians, pain researchers, and a patient partner with lived experience of chronic pain, ensuring alignment with clinical priorities and patient-centred outcomes.

**Figure 2.**
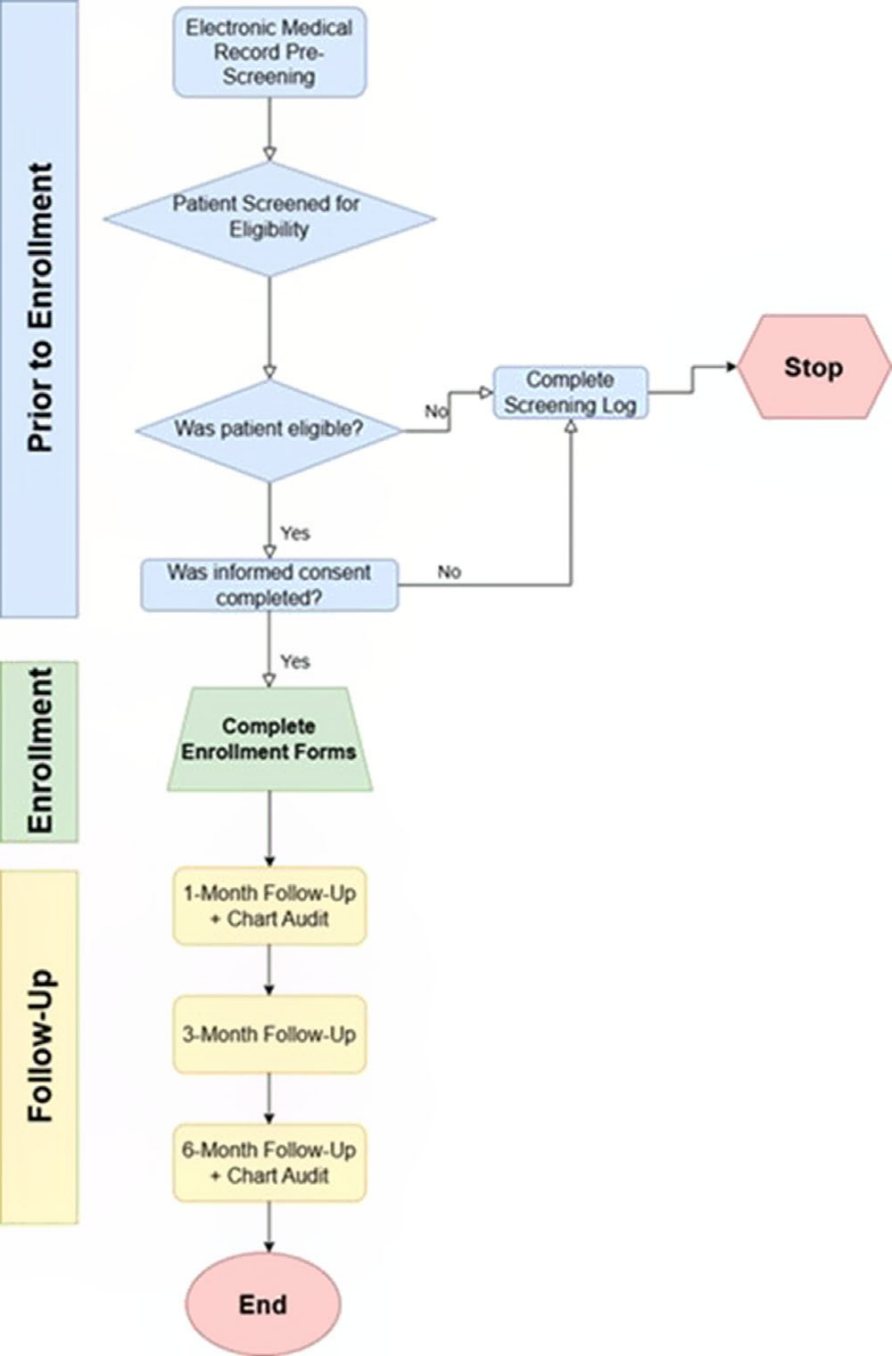
Study Flowchart. Flowchart outlining the PREVENT study procedures for participant screening, enrolment, and follow-up. Potential participants are identified through electronic medical record pre-screening, followed by in-person eligibility assessment and informed consent. Enrolled participants complete baseline forms and are followed at 1-, 3-, and 6-month intervals, with chart audits conducted at the 1- and 6-month time points. Screening logs are completed for all screened individuals regardless of eligibility or consent status.

Recruitment will occur through direct, in-person approaches in the ED based on screening via the electronic medical record (EMR) tracking system (Figure 2).

### Inclusion criteria

Adult ED patients ≥18 years of age will be eligible for participation if they meet specific criteria: (1) have acute neck and/or low back pain (onset ≤4 weeks); (2) have incurred a recent (≤4 weeks) trauma (e.g., motor vehicle crash, falls, bicycle accidents); (3) have no definitive cause of pain related to unstable fracture, malignancy, active infection, or inflammatory conditions (e.g., ankylosing spondylitis); (4) have no co-occurring progressive neurological symptoms (e.g., spinal cord injury or cauda equina syndrome); (5) are not currently pregnant or planning pregnancy within the next 6 months; (6) have no impairments precluding informed consent (e.g., cognitive dysfunction); (7) are English-speaking; and (8) have a willingness to participate in follow-up assessments.

The ≤4-week trauma window ensures that participants are enrolled during the acute phase of pain and that pain onset can be clearly linked to a specific inciting event. This design feature enhances the precision of prospective tracking of pain progression and aligns with established chronic pain definitions, which require symptoms to persist for ≥3 months on most days or every day (Dahlhamer *et al*., 2018; Nahin *et al.*, 2023; Ray *et al.*, 2024). This approach also mirrors methodologies used in post-surgical pain studies, where researchers follow patients from a well-defined index event to assess long-term pain outcomes (Katz & Seltzer, 2009). By anchoring enrolment to a recent traumatic event, we minimize misclassification of subacute or chronic pain and increase the validity of modelling. This approach is consistent with prior ED-based longitudinal research (Beaudoin *et al.*, 2020) and supports reliable identification of acute-to-chronic pain transitions.

### Recruitment

After EMR screening, study staff will coordinate with the patient’s primary ED provider to confirm eligibility and ensure that approaching the patient will not interfere with clinical care. To maintain confidentiality and minimize disruption, recruitment discussions will take place in private or semi-private areas (e.g., examination rooms). Study staff will introduce the study, explaining its purpose, procedures, and voluntary nature, while providing the opportunity for patients to ask questions. If the patient expresses interest, staff will proceed with the informed consent process. Consent will only be obtained once the patient is clinically stable, ensuring that they are in a condition where they can fully comprehend the study details. Participants will be given ample time to review the consent document and ask any questions before signing. Research staff will be available to provide explanations and answer any questions the participant or their family may have. Participants will also have the option to discuss the study with their healthcare provider or family members before deciding. To ensure participants fully understand the research, the research staff will ask comprehension questions after the participant has read the consent form. This step will help assess whether the participant has understood the study, its risk, and benefits. If a participant is unable to demonstrate an understanding of the study, they will not be enrolled. If the treating clinical team or research staff suspects that a patient may have diminished capacity to consent, the patient will not be approached for participation. This ensures that all participants can make an informed decision. Participants will receive a PDF copy of the signed consent form immediately after providing consent via the REDCap (Research Electronic Data Capture) e-consent system. This ensures they have a record of their participation and the study details. All signed consent documents will be securely stored in REDCap. REDCap provides secure electronic storage, ensuring the confidentiality and integrity of participant information in compliance with Health Insurance Portability and Accountability Act (HIPAA) regulations. Since this study is under HIPAA guidelines, all consent documents and related research records will be maintained for a minimum of 6 years following study closure. During this time, the documents will remain securely archived in REDCap, which offers audit trails and data encryption to safeguard participant privacy.

Since January 2025, screening efforts at our primary ED site have identified 217 patients with musculoskeletal pain, of whom 28 met all inclusion criteria, including the ≤4-week trauma requirement. Among those eligible, 25 participants consented and were enrolled, while 3 declined participations, yielding an acceptance rate of approximately 89% and an overall enrolment rate of 11.5% based on screened patients. These preliminary data support the feasibility of enrolling the target sample of 246 participants within the planned 24 – 36-month recruitment period.

### Financial reimbursement

Patient compensation is allocated to incentivize participation, enhance retention, and reduce attrition during the study’s follow-up assessments. Given the longitudinal nature of the study, financial incentives will support continued engagement at key time points, ensuring high-quality data collection and completeness of outcome measures. The distribution of compensation includes:

- Baseline: completion of Neck-Pain & Attitude Questionnaire (Neck-PAQ) validation study: $10
- 1-month follow-up: $50 raffle (four winners; ∼1 in 62 chance)
- 3-month follow-up: $100 raffle (four winners; ∼1 in 62 chance)
- 6-month follow-up: $200 raffle (one winner; ∼1 in 246 chance)

Compensation will be provided through prepaid debit cards or electronic payment methods, ensuring timely and convenient distribution to participants. The incentive structure aligns with best practices for participant retention in longitudinal studies, while remaining compliant with NIH and institutional research (Teague *et al.*, 2018).

### Quantitative outcomes & assessment tools

To ensure standardization and data harmonization with other studies, this study aligns with the HEAL Common Data Elements (CDE) repository (Wandner *et al.*, 2021). Key measures, including pain intensity (VAS), pain frequency (NHIS Pain Chronicity), psychological assessments (PCS, TSK, PHQ-2, GAD-2), functional status (ODI, NDI), and substance use screening (ASSIST), were selected from HEAL’s CDE repository whenever possible (Table 1). Participant follow-up assessments will be conducted at 1-, 3-, and 6-months post-ED visit to track pain progression and related outcomes.

**Table 1.** Study measures & assessment tools

Domain	Instrument	Description	Time	Analysis Role
Pain Frequency	Adaptation of National Health Interview Survey (NHIS) Pain Chronicity Question (Duca *et al.*, 2022)	Longitudinal assessment of pain frequency.	0, 1, 3, 6 months	Primary Outcome
Pain Intensity	Visual Analogue Scale (VAS) (Kelly, 2001)	Quantitative metric of an individual’s pain on a numerical scale.	0, 1, 3, 6 months	Secondary Outcome
Functional Status (Low Back)	Oswestry Disability Index (ODI) (Yoshida *et al.*, 2019)	Assesses disability related to low back pain.	0, 1, 3, 6 months	Secondary Outcome
Functional Status (Neck)	Neck Disability Index (NDI) (Pool *et al.*, 2007)	Assesses disability related to neck pain.	0, 1, 3, 6 months	Secondary Outcome
Pain Beliefs	Back/Neck-Pain Attitude Questionnaire (B-PAQ/N-PAQ) (Darlow *et al.*, 2014)	Assesses pain beliefs and attitudes related to neck and back pain.	0, 1, 3, 6 months	Independent Variable
Pain Self-Efficacy	Pain Self-Efficacy Questionnaire-2 (PSEQ-2) (Nicholas et al., 2015)	Assesses an individual’s confidence in performing daily activities despite experiencing pain.	0, 1, 3, 6 months	Mediator
Recovery Expectations	Expectation of Recovery (Single-item measurement) (Carrière *et al.*, 2023)	Assesses individual’s expectation for recovery from current pain episode.	0, 1, 3, 6 months	Mediator
Fear-Avoidance Behaviour	Tampa Scale of Kinesiophobia (TSK) (Tkachuk & Harris, 2012)	Quantitative metric asking 11 questions using a 4-point Likert scale regarding a participant’s fear and avoidance of movement.	0, 1, 3, 6 months	Mediator
Catastrophic Thinking	Pain Catastrophizing Scale (PCS) (Monticone *et al.*, 2022)	13-item quantitative metric assessing participant’s catastrophic thinking about their pain experience, contributing to rumination, magnification, and learned helplessness.	0, 1, 3, 6 months	Mediator
Physical Activity	International Physical Activity Questionnaire-Short Form (IPAQ-SF) (Oliveira *et al.*, 2023)	Assesses self-reported physical activity levels over the past 7 days, including walking, moderate-intensity, and vigorous-intensity activities, as well as sedentary time.	0, 1, 3, 6 months	Mediator
Anxiety	Generalized Anxiety Disorder-2 (GAD-2) (Sapra *et al.*, 2020)	Brief screening tool for generalized anxiety disorder.	0, 1, 3, 6 months	Covariate
Depression	Patient Health Questionnaire-2 (PHQ-2) (Kroenke *et al.*, 2003)	Brief screening tool for depressive symptoms.	0, 1, 3, 6 months	Covariate
Substance Use	Alcohol, Smoking, and Substance Involvement Screening Test (ASSIST) (WHO Assist Working Group, 2002)	Screens for substance use risk, including alcohol, tobacco, and drug use.	0, 1, 3, 6 months	Covariate
Social Determinants of Health (SDOH)	Accountable Health Communities (AHC) Health-Related Social Needs (HSRN) Screening Tool from Centers for Medicare & Medicaid Services (CMS) (Centers for Medicare & Medicaid Services, 2021)	Evaluates social factors affecting health, including financial strain, employment, housing, food security, transportation, and social support.	0, 1, 3, 6 months	Covariate
Demographics	N/A	Age, Sex, Insurance, Co-Morbidities, Race/ethnicity, Past Medical History	0	Covariate

The primary outcome is the development of CMP in the neck and/or low back, defined as pain in the same anatomical region as the acute presenting complaint, present on most days or every day for at least three months. Secondary outcomes will include changes in pain beliefs (assessed through B-PAQ/N-PAQ scores), pain catastrophizing (PCS scores), and fear-avoidance behaviours (TSK scores), and healthcare utilization, including recurrent ED visits and outpatient care. Additional secondary measures will evaluate changes in physical activity levels (IPAQ-SF scores), pain self-efficacy (PSEQ-2 scores), and pain-related disability, measured through changes in ODI and NDI scores over time. While this study applies the Cognitive Risk Model for CMP specifically to post-traumatic neck and/or low back pain, the model is designed to be adaptable to other anatomical regions and chronic pain types – including both chronic primary and secondary musculoskeletal pain – in future research (Treede *et al.*, 2015).

In addition to baseline enrolment forms, we will perform a chart audit post-ED discharge to assess ED management, including ED imaging (x-ray, CT, MRI), medication management (including opioids, NSAIDs, acetaminophen, and muscle relaxants), ED disposition (admission vs discharge), diagnostic codes (ICD-10 for musculoskeletal and neurological conditions), and referrals provided (e.g., primary care, pain specialist, physical therapy). After 1- and 6-month follow-ups, additional audits will be completed utilizing an automated process to query the EMR using the Financial Identification Number (FIN) and Medical Record Number (MRN) from the index ED visit. Using these patient-specific identifiers collected during enrolment, a report can be generated for pre-specified data fields in the EMR which exactly matches the data dictionary in the case report form (CRF) built in REDCap. Data can be imported into REDCap and will be checked for fidelity and missingness. An advantage of this process is reduction in bias inherent in manual chart review (Gilbert *et al.*, 1996). As a further check, we will query the regional health information exchange for relevant healthcare utilization at regional outside hospitals.

### Sample size & quantitative data analysis

Our primary outcome – pain frequency – is assessed as a categorical variable based on participants’ reported number of days per week experiencing neck or low back pain (0–1 days, 2–3 days, 4–5 days, or 6–7 days). Pain frequency will be assessed at 1, 3, and 6 months. For analysis, responses will be dichotomized to classify CMP status: participants reporting pain on 4–5 or 6–7 days per week will be categorized as having CMP (coded as 1), while those reporting 0–1 or 2–3 days per week will be categorized as not having CMP (coded as 0). Based on prior studies reporting a 12%–27% transition rate from acute to chronic pain (Beaudoin *et al.*, 2020; Office of the Assistant Secretary for Planning and Evaluation, 2021; Bourassa *et al.*, 2020; Barnett *et al.*, 2017), we conducted a power analysis to estimate the incidence of CMP with acceptable precision. Using a small effect size (Cohen’s d 0.2), with α = 0.05 and power = 0.80, a minimum sample size of 197 participants is required. To account for an estimated 25% attrition rate, we will recruit 246 participants to ensure adequate study power. This sample size will also provide sufficient power to explore the association between baseline pain beliefs and subsequent development of CMP, as well as to conduct preliminary analyses for establishing potential clinical cut-offs on pain relief measures. Higher scores may indicate individuals at elevated risk for developing CMP and who may benefit from targeted educational interventions. However, these exploratory analyses will require validation in independent samples.

Quantitative statistical analysis will involve predictive model development using multivariable logistic regression to identify significant predictors of CMP as a binary outcome. Pain beliefs will be modelled as the primary independent variable, with adjustment for potential confounding variables identified in the conceptual framework (e.g., demographics, anxiety, depression, substance use, and social determinants of health). Mediation analyses will explore indirect pathways through cognitive-affective constructs (e.g., pain catastrophizing, fear-avoidance, recovery expectations, and pain self-efficacy) and behavioural responses (e.g., physical activity), using structural equation modelling or mediation analysis to estimate direct and indirect effects. Interaction terms between pain beliefs and demographic variables will be tested to evaluate potential effect modification. Model validation will incorporate cross-validation techniques, such as k-fold cross-validation, to assess robustness. Receiver operating characteristic (ROC) curve analysis will determine the model’s discriminative ability using area under the curve (AUC) estimates, and calibration analysis (e.g., Hosmer–Lemeshow test) will assess model fit. Preliminary clinical cut-offs for pain beliefs scores will also be explored by identifying thresholds that maximize sensitivity and specificity for predicting CMP developed based on ROC analysis. Sensitivity analyses will evaluate the stability of findings under alternative variable definitions and measurement criteria.

### Validation of the neck-pain and attitudes questionnaire (Neck-PAQ)

During patient screening in the ED, individuals presenting with acute neck pain will be identified as potential participants for the Neck-PAQ validation study. Eligible and consenting participants will be invited to complete the Neck-PAQ and participate in a Think Aloud interview to evaluate the tool’s clarity, interpretability, and response process validity (Scott *et al.*, 2021). The Think Aloud methodology involves asking participants to verbalize their thoughts in real-time as they complete the questionnaire (Padilla & Leighton, 2017). This process enables the research team to assess how respondents interpret and process each item, and whether the intended constructs are being accessed during response formulation. Participants will be reminded that the goal is to capture their natural reasoning and will complete a short practice session to increase comfort with the process (Padilla & Leighton, 2017). All Think Aloud interviews will be audio-recorded, securely stored in REDCap and assigned an identification number. Audio recordings will be transcribed using Whisper (OpenAI, 2023), an automated speech recognition system that operates locally without requiring internet access, thereby minimizing risks to protected health information (PHI). Manual verification will be conducted to ensure transcription accuracy. Anonymized transcripts will then be uploaded to NVivo (QSR International Pty Ltd, 2020) for qualitative analysis. The original audio files will be securely stored on password-protected drives accessible only to study personnel. Upon study completion, all audio recordings will be permanently deleted. Audio will be used solely for data analysis and will not be included in any presentation, publication, or non-research setting. Only transcribed, de-identified excerpts will be used for dissemination purposes.

### Qualitative sample size & data analysis

**Figure 3.**
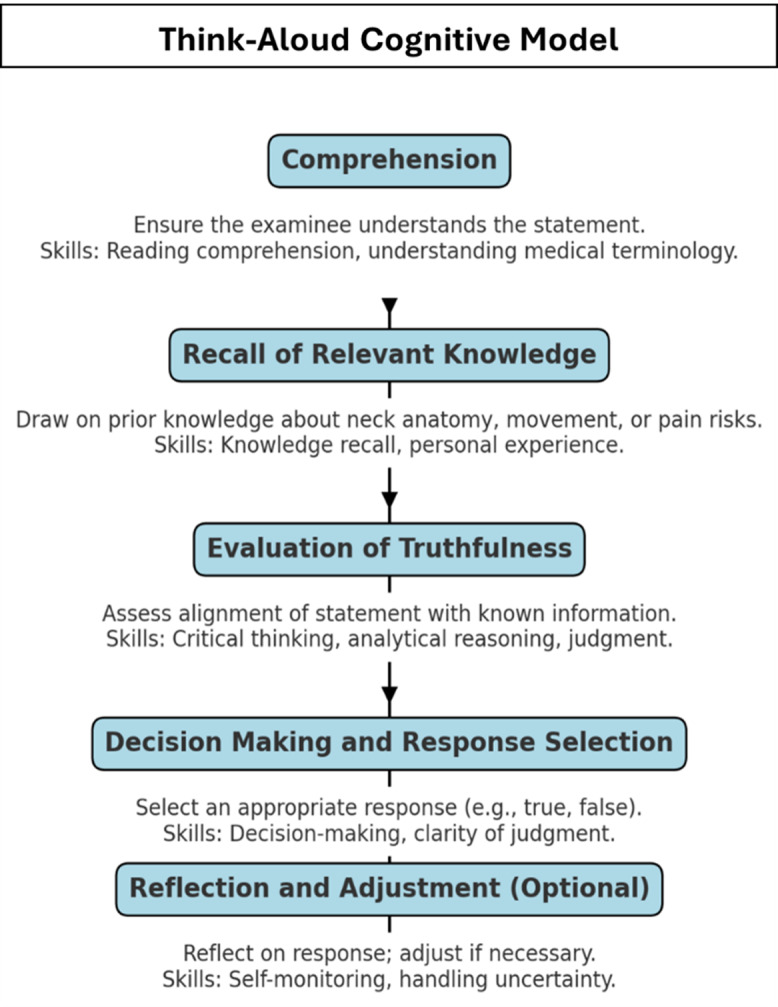
Think Aloud Cognitive Model. This model outlines the cognitive processes engaged by participants during the Think Aloud protocol, including comprehension, recall of relevant knowledge, evaluation of truthfulness, decision making and response selection, and optional reflection and adjustment. Each stage highlights key cognitive skills involved, such as reading comprehension, analytical reasoning, and judgement, used to interpret and respond to pain-related questionnaire items.

The target sample size is guided by the concept of information power, which considers the study aim, sample specificity, theoretical grounding, quality of dialogue, and chosen analytical strategy (Malterud *et al.*, 2016). As the Neck-PAQ is a newly developed instrument and the aim is narrow and well-defined, a sample of approximately 20 participants is expected to yield sufficient information power to evaluate the appropriateness of the tool for capturing maladaptive beliefs about the neck, pain, management, and recovery. Verbal data from the Think Aloud interviews will be analysed using deductive content analysis guided by a predefined cognitive model of response processes (Figure 3). This model includes five domains: Comprehension, Knowledge Recall, Evaluation of Truthfulness, Decision-Making and Response Selection, and Reflection and Adjustment (see appendix Neck-PAQ Coding Manual). Each distinct thought unit will be assigned to one or more of these categories. Coding will be conducted independently by two trained raters. Inter-rater reliability will be calculated using Cohen’s Kappa, with a threshold of ≥0.60 required to indicate acceptable agreement (McHugh, 2012).

Discrepancies will be discussed and resolved collaboratively, with updates to rater training or the coding manual as necessary. Although this is a deductive process, qualitative insights may emerge during analysis. These will be descriptively reported, but the core analytic strategy focuses on the structured categorization of verbalized response processes to validate whether items in the Neck-PAQ are functioning as intended.

## Stakeholder engagement

The development and refinement of this study were informed by input from multiple stakeholder groups. ED clinicians, pain researchers, and a patient partner contributed to the design phase, providing feedback to ensure clinical feasibility, contextual relevance, and representation of the lived experience of chronic pain. The patient partner will also contribute to the qualitative data analysis, interpretation of findings, and dissemination of results. Stakeholder involvement enhances the study’s relevance, credibility, and potential impact by ensuring that research questions, methods, and outcomes reflect patient-centred priorities and real-world clinical needs, while minimizing patient blaming and stigmatization (Ray *et al.*, 2024; Cohen *et al.*, 2011).

## Ethical & safety considerations

This study poses minimal risk to participants; however, several protections are in pace to address potential concerns. The primary risk involves the potential loss of confidentiality. Although all study data will be stored securel using HIPAA-compliant REDCap servers with encryption and strict access controls, a small risk of data breach remains. To mitigate this, no PHI will be stored alongside study data, and all identifiable information will be de-identified prior to analysis. Data elements will be clearly labelled as PHI or non-PHI to ensure regulatory compliance.

There are no physical risks associated with this study, as participation involves only surveys and interviews. Psychological or emotional discomfort may occur, particularly when answering questions related to pain, mental health, or substance use. Participants will be informed that they may skip any question or withdraw from the study at any time without penalty or impact on their medical care.

Written informed consent will be obtained from all participants, with clear explanation of study procedures, risks, and confidentiality safeguards. In alignment with mandated reporting procedures, if a participant discloses child or elder abuse, neglect, or an imminent threat of harm such as suicidal or homicidal ideation, the research staff will notify the ED care team and the study’s PI, who will then follow institutional reporting guidelines.

Participants will have access to mental health support throughout the study. Since the study is conducted within the ED, patients will have 24/7 access to social workers and psychological consultations as needed. Participants will also be provided with information about external mental health resources, including the university’s Employee Assistance Program (EAP), student counselling services, and national crisis support lines such as the 988 Suicide & Crises Lifeline.

## Data management & dissemination

Study data, including de-identified clinical variables, patient-reported outcomes, and qualitative transcripts, will be stored on HIPAA-compliant servers and shared via NIH-compliant repositories such as Vivli. Access to individual-level data will require data use agreements, while metadata, survey instruments, and analytic code (SAS, R, Python) will be provided to support transparency and reproducibility. Oversight will be maintained by the study PI in collaboration with the IRB and institutional data support teams.

## Discussion

Musculoskeletal pain – particularly in the neck and lower back – is the leading cause of disability worldwide and a frequent reason for ED visits (Vos *et al.*, 2020; World Health Organization, 2022; Centers for Disease Control and Prevention, 2023). Despite its high in acute care settings, few studies have systematically investigated early predictors of chronicity or developed target interventions during this critical window. The Pain Recognition and Evaluation to Validate Effective Neck and back Treatment (PREVENT) study addresses this gap by prospectively evaluating cognitive, behavioural, and contextual factors that may contribute to the transition from acute to chronic pain following traumatic injury.

Chronic pain affects approximately 1 in 4 adults and 1 in 5 children, contributing to increased rates of co-morbid health conditions and increased mortality risk. (Dahlhamer *et al*., 2018; Chambers *et al.*, 2024; Foley *et al.*, 2021; Ray *et al.*, 2024). Individuals with chronic pain experience double the risk mortality compared to those without, and those with high-impact chronic pain – defined as chronic pain that limits life or work activities – face a 2.5-fold increased risk (Dahlhamer *et al*., 2018; Ray *et al.*, 2024). Among individuals living with chronic pain, an estimated 8%–12% meet criteria for opioid use disorder, and many also have elevated odds of other substance use disorders (Vowles *et al.*, 2015; Havlik *et al.*, 2024). Early identification of high-risk individuals, coupled with scalable, evidence-based interventions, is essential to interrupt this trajectory. Findings from this study may inform ED-based strategies to reduce the incidence of CMP, improve long-term health outcomes, and decrease healthcare utilization and opioid-related harms.

One promising framework is the Screening, Brief Intervention, and Referral to Treatment (SBIRT) approach, which has been successfully applied to substance use and mental health conditions but has not yet been adapted for acute pain management (Barata *et al.*, 2017; Hargraves *et al.*, 2017). In musculoskeletal pain scenarios, guideline-concordant care emphasizes a person-centred approach that includes education, shared decision making, and supported self-management (Lin *et al.*, 2020; Hutting *et al.*, 2022).

Pain science education (PSE) is an evidence-based intervention designed to support individuals seeking care for musculoskeletal pain (Watson *et al.*, 2019). PSE helps patients reconceptualize pain away from being viewed solely as a marker of tissue damage or injury (more pain equals more damage) to that of a protective mechanism. In this way, PSE provides a scientific basis for a BioPsychoSocial model of pain and disability and the enhanced sensitivity generated by central nervous system adaptations as pain persists (Moseley & Butler, 2015). PSE consists of providing people with information about the various contributors to pain, the meaning of pain, and supported self-management strategies (Moseley & Butler, 2105). High-level evidence from randomized controlled trials have demonstrated positive effects of PSE on disability and fear of movement (Watson et al., 2919). Further, recent evidence identified target concepts important to individuals’ recovery from musculoskeletal pain and included narratives such thoughts, emotions, and experiences affect pain (Leake *et al.*, 2021). However, to date, PSE interventions have not been adapted for delivery in an ED setting. SBIRT offers a validated framework for embedding PSE and facilitating referrals for treatment in acute pain scenarios, particularly for patients identified at high-risk for CMP via a cognitive risk model.

Ongoing research is adapting SBIRT in an outpatient setting to encourage veterans to seek healthcare for musculoskeletal pain-related disorders through non-pharmacological interventions while also addressing co-occurring mental health and substance use issues (Martino *et al.*, 2020). This provides preliminary evidence that SBIRT can be adapted for pain management scenarios. Building on this work, our planned investigations will adapt SBIRT for acute musculoskeletal pain in the ED setting – an approach we refer to as *Pain-SBIRT*. In this approach, *Pain-SBIRT* will incorporate real-time delivery of PSE during the ED consultation and include follow-up assessments after discharge. Patients identified as high-risk through our predictive model will be prioritized, ensuring that intervention resources are directed to those most vulnerable to pain chronicity. These innovations position Pain-SBIRT as a scalable, patient-centred intervention for acute pain in ED settings.

Finally, while this study focuses on post-traumatic neck and back pain, the underlying Cognitive Risk Model is designed to be adaptable across different anatomical regions and applicable to both primary and secondary musculoskeletal pain syndromes (Treede *et al.*, 2015). Future research may extend its utility to broader populations or settings, such as paediatric care or underserved community clinics, enhancing the model’s translational potential and public health impact.

## Conclusion

This research will advance our understanding of the biopsychosocial factors influencing the transition from acute to chronic neck and back pain, particularly within the ED setting. By identifying modifiable psychological and behavioural risk factors – such as maladaptive pain beliefs, fear-avoidance, and low self-efficacy – this study will lay the groundwork for the development of targeted, evidence-informed interventions. Specifically, findings may inform the adaptation of existing frameworks, such as SBIRT for pain management and enable the tailored delivery of PSE to high-risk patients. These approaches could reduce recurrent ED visits, improve long-term patient outcomes, and support the integration of preventative strategies into clinical guidelines and health policy. As a foundational study, this work will also support larger-scale trials and public health efforts to reduce the burden of CMP.
